# Artificial intelligence model predicts M2 macrophage levels and HCC prognosis with only globally labeled pathological images

**DOI:** 10.3389/fonc.2024.1474155

**Published:** 2024-12-20

**Authors:** Huiyuan Tian, Yongshao Tian, Dujuan Li, Minfan Zhao, Qiankun Luo, Lingfei Kong, Tao Qin

**Affiliations:** ^1^ Department of Scientific Research and Foreign Affairs, Henan Provincial People’s Hospital, Zhengzhou University People’s Hospital, Zhengzhou, Henan, China; ^2^ School of Computer Science and Technology, University of Science and Technology of China, Hefei, Anhui, China; ^3^ Department of Pathology, Henan Provincial People’s Hospital, Zhengzhou University People’s Hospital, Zhengzhou, Henan, China; ^4^ Department of Hepatobiliary and Pancreatic Surgery, Henan Provincial People’s Hospital, Zhengzhou University People’s Hospital, Zhengzhou, Henan, China

**Keywords:** deep learning, masked autoencoders, computational pathology, liver cancer, tumor microenvironment

## Abstract

**Background and aims:**

The levels of M2 macrophages are significantly associated with the prognosis of hepatocellular carcinoma (HCC), however, current detection methods in clinical settings remain challenging. Our study aims to develop a weakly supervised artificial intelligence model using globally labeled histological images, to predict M2 macrophage levels and forecast the prognosis of HCC patients by integrating clinical features.

**Methods:**

CIBERSORTx was used to calculate M2 macrophage abundance. We developed a slide-level, weakly-supervised clustering method for Whole Slide Images (WSIs) by integrating Masked Autoencoders (MAE) with ResNet-32t to predict M2 macrophage abundance.

**Results:**

We developed an MAE-ResNet model to predict M2 macrophage levels using WSIs. In the testing dataset, the area under the curve (AUC) (95% CI) was 0.73 (0.59-0.87). We constructed a Cox regression model showing that the predicted probabilities of M2 macrophage abundance were negatively associated with the prognosis of HCC (HR=1.89, p=0.031). Furthermore, we incorporated clinical data, screened variables using Lasso regression, and built the comprehensive prediction model that better predicted prognosis. (HR=2.359, p=0.001).

**Conclusion:**

Our models effectively predicted M2 macrophage levels and HCC prognosis. The findings suggest that our models offer a novel method for determining biomarker levels and forecasting prognosis, eliminating additional clinical tests, thereby delivering substantial clinical benefits.

## 1 Introduction

According to the Global Cancer Statistics 2020, liver cancer is the third leading cause of cancer-related mortality, accounting for 8.3% of deaths globally, and the seventh most commonly diagnosed malignancy, comprising 4.7% of all cancer cases, causing serious public health problems and economic burden (1).

Tumor-associated macrophages (TAMs), which originate from bone marrow-derived blood monocytes (monocyte-derived macrophages) or yolk sac progenitors (tissue-resident macrophages), are a specialized subset of immune cells that are abundantly present in the tumor microenvironment of various solid tumors (2). There are two polarization types of TAMs. Under the stimulation of lipopolysaccharide (LPS), IFN-γ, etc., TAMs are activated into classically activated macrophages (M1 type). Conversely, under the stimulation of anti-inflammatory factors such as IL-10, TGF-β, etc., TAMs are activated into alternatively activated macrophages (M2 type) (3). Previous research indicates that M2 macrophages play a critical role in cancer-related inflammation and are involved in various aspects of tumor biology, including immunosuppression, tumor progression, invasion, and metastasis (2, 4–8).

CIBERSORTX, a bioinformatics tool developed by the Alizadeh Lab and Newman Lab, can be used to estimate the abundance of various cell types in a mixed cell population based on gene expression data (4). However, this approach relies on transcriptome sequencing, which is not a commonly performed clinical test and can be expensive. In recent years, image recognition technology has become increasingly prevalent in the medical field. Pathological tissues are routinely collected during the diagnosis and treatment of patients undergoing HCC surgery, and Whole Slide Images (WSIs) can provide valuable insights into tumor heterogeneity. Predicting the relative abundance of M2 macrophages directly from WSI images and subsequently forecasting patient survival would not only be more cost-effective and resource-efficient than the CIBERSORTx method but also highly convenient and promotable.

Implementing supervised learning in deep neural networks requires a substantial amount of labeled data. However, manual data labeling is both time-consuming and costly. Additionally, acquiring sufficient data in the medical field remains a significant challenge. WSIs typically contain more than 100,000 x 100,000 pixels (9), rendering them impractical for direct processing by convolutional neural networks (CNNs). The conventional approach involves dividing a WSI into patch-level images, often numbering over 10,000, and then individually annotating each image. However, this process also demands considerable human effort. Some studies have attempted to address this issue using weakly supervised clustering methods. The main strategies include increasing the sample size of the training set (10) and employing transfer learning techniques (11).

Therefore, the primary bottleneck in current computational pathology is the annotation of labels and the limited quantity of samples. The key technology employed in this study is the Masked Autoencoder (MAE), a self-supervised autoencoder that masks a portion of the input image and attempts to predict the masked tiles based on the visible tiles (12).

While self-supervised learning methods are widely used in natural language processing (NLP) (13, 14), the field of computer vision (CV) predominantly relies on supervised methods. Recent research by Kaiming He and colleagues has demonstrated that for visually dense images with high information redundancy, constructing a challenging task by masking a substantial proportion of patches can yield results comparable or even superior to those of supervised training (12).

In our study, we introduce a novel MAE-ResNet modeling method that integrates MAE with ResNet-32t, presenting a slide-level, weakly-supervised clustering approach for WSIs. Based on this, we developed and validated models to determine M2 macrophage levels and further predict patient prognosis.

## 2 Methods

### 2.1 Datasets

The data used in this study were sourced from Henan Provincial People’s Hospital and The Cancer Genome Atlas (TCGA) database, a comprehensive multi-center repository containing genomic data, clinical information, and pathological slides from various hospitals worldwide (15). The HCC cases from both sources contained information such as age, gender, stage, grade, T, N, M, surgery type, and overall survival. On this basis, the inclusion criteria for our study were: 1) pathologically diagnosed HCC, and 2) availability of at least one formalin-fixed and paraffin-embedded slide. The exclusion criteria were: 1) metastatic liver cancer, and 2) concurrent malignancies. For the TCGA dataset, in addition to the above criteria, RNA sequencing data were also required. Ultimately, the study included 132 HCC patients from the hospital and 353 from the TCGA database. This study aims to establish two models: one model to predict the relative abundance of M2 macrophages and another to forecast the prognosis of HCC patients using WSIs. The TCGA dataset is used to build and validate M2 macrophage level prediction model, while both the TCGA and hospital datasets are used for prognosis prediction.

### 2.2 Calculation of the relative abundance of M2 macrophages

We first obtained the RNA sequencing profile of HCC from TCGA database and downloaded the corresponding gff3 file (http://ftp.ebi.ac.uk/pub/databases/gencode/Gencode_human/release_22/gencode.v22.anno tation.gff3.gz) from GENCODE (https://www.gencodegenes.org/human/). Using gene length and gene count data, we calculated the Transcripts Per Million (TPM) for each gene. We employed CIBERSORTx to calculate the relative abundance of M2 macrophages. The tool is available at https://cibersortx.stanford.edu/. LM22 (22 immune cell types) was used as signature matrix file. We categorized the relative abundance of M2 macrophages into two groups: high and low. Subsequently, we developed a ResNet model, which was trained and validated to effectively distinguish between high and low relative abundances of M2 macrophages.

### 2.3 MAE-based weakly supervised learning method

**Figure 1:**
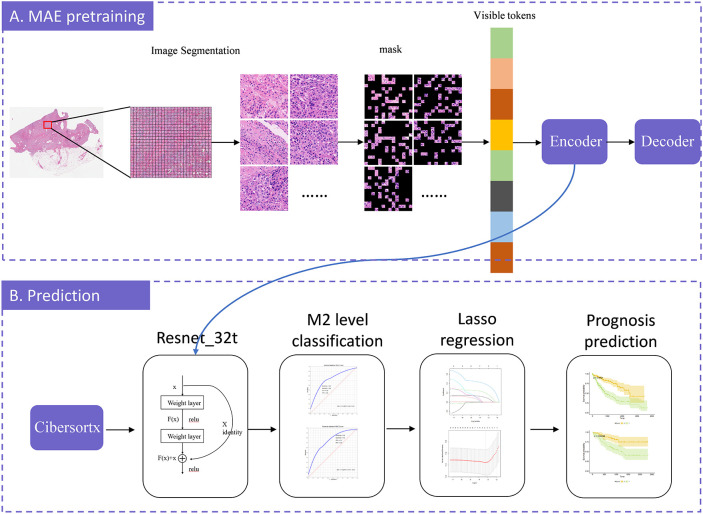
The model-training process illustration. The model construction consists of two stages: MAE-pretraining and prediction. In the MAE-pretraining phase, we first divided each slide into patches, and then split each patch into tiles. We randomly masked a portion of the tiles and predicted these masked tiles. The model was continuously optimized by minimizing the difference between the predicted tiles and the original image, iteratively improving prediction accuracy. Once the optimal prediction model was identified, we exported the encoder component to serve as the input for ResNet-32t. We employed CIBERSORTx to calculate the relative abundance of M2 macrophages and, based on these calculations, established a model to predict the relative abundance of M2 macrophages from WSI. We further employed LASSO regression to select potential prognostic factors and, by combining these factors with the predicted relative abundance of M2 macrophages, predicted overall survival. Finally, the model was validated using an external dataset.

MAE is a self-supervised autoencoder. We integrated MAE with ResNet-32t to introduce a slide-level, weakly-supervised clustering method for WSIs in order to predict the relative abundance of M2 macrophages. MAE demonstrates strong performance when a significant proportion of the input images are masked (12). Building on this, we utilized the encoder component of the MAE architecture to encode pathological images. These encoded images were then used to classify the relative abundance of M2 macrophages and to predict survival rates, using slide-level labels. In this M2 macrophage level prediction model, we randomly divided the TCGA dataset into training, validation, and testing sets in a 7:1.5:1.5 ratio. The overall training process was illustrated in **Figure 1**. The code for our study is available on GitHub (https://github.com/MinfanZhao/M2-HCC-Prognosis).

#### 2.3.1 Pre-processing

In the image preparation stage, we initially performed color standardization using histogram equalization. Due to the large size of WSI images and video memory limitations, they cannot be directly processed within the model. To address this issue, we segmented the images into smaller patches and resize them to 512 × 512 pixels. Additionally, we discarded patches containing more than 50% background areas.

#### 2.3.2 Model training

The model training process comprises two components: MAE pre-training and ResNet-32t.

MAE pre-training can be divided into two asymmetrical components: an encoder and a decoder. The encoder was essentially a Visual Transformer (VIT) network, and in this study, the adopted model was ViT-large. In ViT-large, a random section of the input image was masked, and subsequently, the missing pixels were reconstructed. Each patch was further resized into tiles. We randomly shuffled these tiles, selected 25% for encoding, and masked the remaining 75%. Positional embeddings, along with 25% of the encoded tiles, are fed into the decoder to maintain the original order of the image blocks. The decoder then attempted to reconstruct the original image using the input information provided. Ultimately, the output image from the decoder was compared with the original image to compute the error. This error was backpropagated to update the model’s parameters. Through extensive training with WSI patches, the artificial intelligence model learned the information within the images and reconstructs them, thereby completing the MAE model training.

Subsequently, we retained only the encoder’s output, which consists of 1024-dimensional vectors, fed these into ResNet-32t to obtain the final classification results. This transformation enables the simultaneous loading of all WSI patches into GPU memory. Considering “Cluster High” as the positive class, our trained ResNet-32t predicted and categorized all slides as either “Cluster High” or “Cluster Low”.

#### 2.3.3 Model testing

We used the validation dataset to assess the classification performance of the proposed method for weight selection during ResNet-32t training. We evaluated the AUC (Area Under the Curve) of our method by comparing the predicted levels of M2 macrophages in the testing dataset with the ground truth from liver histopathological slides.

Additionally, we calculated the ROC curves and AUC for classifying the relative abundance of M2 macrophages using the “pROC” package in R. We also computed the sensitivity, specificity, PPV (Positive Predictive Value), and NPV (Negative Predictive Value) for the optimal cut-off value and visualized the results using the ggplot2 package in R.

### 2.4 Prognosis prediction

Given the well-documented correlation between the relative abundance of M2 macrophages and tumor prognosis, we used the predicted probabilities of M2 macrophage levels (M2prob) from our MAE-ResNet model to predict the overall survival of HCC patients. Additionally, we aimed to develop a prognostic model using M2prob in conjunction with clinical data. In this prognosis prediction model, we used the TCGA dataset as the training set and the hospital data as the testing set. All statistical analyses were performed using R version 4.2.3.

#### 2.4.1 Data cleaning and clinical features

The independent variables included in the analysis were T (the range and size of the primary tumor), N (lymph node metastasis), M (the presence of metastasis), age, gender, stage, grade, surgery, overall survival, and event. General clinical information of the subjects and statistical tests for differences between the training and testing datasets are presented in **Table 1**. We performed t-tests, Wilcoxon rank-sum tests, chi-squared tests, or Fisher’s Exact Test, depending on the data type. Shapiro-Wilk tests were conducted for continuous variables to assess normality. For variables that met the criteria for normal distribution, t-tests were used; for those that did not, Wilcoxon rank-sum tests were employed. For categorical variables, Fisher’s exact test or chi-square tests were applied based on theoretical frequencies; for ordinal variables, the Wilcoxon rank-sum test was used. For missing values, multiple imputation was employed to fill the gaps. **Table 1** displays the original data prior to imputation.

**Table 1:** The clinical features of the HCC patients.

	TCGA dataset	Hospital dataset	statistics	*p*
Age	61.00 (51.00,68.25)	57.00 (49.50,66.00)	W = 18867^1^	***0.002***
Gender			χ^2^ = 1.42^2^	0.234
Male	236 (66.86%)	95 (72.52%)		
Female	117 (33.14%)	36 (27.48%)		
Stage^3^			W = 22212^1^	0.192
Stage I	163 (49.24%)	47 (37.60%)		
Stage II	81 (24.47%)	49 (39.20%)		
Stage III	83 (25.08%)	26 (20.80%)		
Stage IV	4 (1.21%)	3 (2.40%)		
Grade^4^			W = 23719^1^	0.198
G1	48 (13.75%)	6 (4.72%)		
G2	167 (47.85%)	70 (55.12%)		
G3	123 (35.24%)	47 (37.01%)		
G4	11 (3.15%)	4 (3.15%)		
T^5^			W =23610^1^	0.1748
T1	172 (49.00%)	47 (37.60%)		
T2	88 (25.07%)	50 (40.00%)		
T3	77 (21.94%)	19 (15.20%)		
T4	14 (3.99%)	9 (7.20%)		
N^6^			Fisher’s Exact Test	1
N0	244 (98.79%)	131 (99.24%)		
N1	3 (1.21%)	1 (0.76%)		
M^7^			Fisher’s Exact Test	1
M0	257 (98.47%)	130 (98.48%)		
M1	4 (1.53%)	2 (1.52%)		
Surgery			W=15360^1^	***<0.001***
Segmentectpmy	32 (12.60%)	8 (8.42%)		
Lobectomy	53 (20.87%)	55 (57.89%)		
Ectended Lobectomy	169 (66.54%)	32 (33.68%)		
Overall survival	601 (348,1189)	919.5 (729.5,1329.5)	W = 20879	***<0.001***
Event			χ^2^ = 0.181	0.670
Alive	226 (64.20%)	60 (61.86%)		
Dead	126 (35.80%)	37 (38.14%)		

^1.^Wilcoxon rank-sum tests were performed.

^2.^Chi-square test was performed.

^3^ Stage group based on the American Joint Committee on Cancer (AJCC) 8^th^ Edition staging criteria.

^4.^The levels of the degree of tumor differentiation. G1~G4 represent the degree of differentiation getting worse and worse.

^5.^The size of the primary tumor according to AJCC 8^th^ staging criteria.

^6.^The defined absence (N0) or presence (N1) of lymph node metastasis according to the AJCC 8^th^ staging criteria.

^7.^The defined absence (M0) or presence (M1) of distant spread or metastases according to the AJCC 8^th^ staging criteria. Bold values indicate p-values less than 0.05.

#### 2.4.2 Relationship between M2prob and prognosis

We utilized a Cox regression model to assess the association between M2prob and the prognosis of HCC patients in the TCGA dataset, subsequently validating these findings in the hospital dataset. Cox regression analysis was conducted using the “survival” package in R.

#### 2.4.3 Survival prediction model construction

General clinical information is closely associated with the prognosis of HCC. We contemplated the integration of general clinical information and M2prob to establish a prediction model. Lasso regression was employed to preliminarily screen all variables presented in **Table 1**, and the model with the smallest mean squared error (MSE) was selected. Subsequently, we used this model to calculate the Mscore, predicted the prognosis of HCC, and verified it in the validation set.

### 2.5 Ethical declaration

Ethical approval for this study was granted by the Ethical Committee of Henan Provincial People’s Hospital in 2022. All procedures performed in this study adhered to the ethical standards of both the institutional and national research committees, as well as the Declarations of Helsinki and Istanbul.

## 3 Results

### 3.1 Clinical features

In the prognosis prediction section, TCGA patients were employed for model training, and patients from Henan Provincial People’s Hospital were used for validation. The following clinical variables were tested: age, gender, stage, grade, T (tumor size), N (lymph node involvement), M (metastasis presence), surgery, overall survival, and event status). The testing results are presented in **Table 1**. In the TCGA dataset, the median age of study subjects was 61 years (interquartile range: 51.00-68.25), with an median overall survival of 601 days (interquartile range: 348-1189) and an outcome event incidence rate of 35.80%. In the hospital dataset, the median age of the study subjects was 57 years (interquartile range: 49.50-66.00), with an median overall survival of 919.5 days (interquartile range: 729.5-1329.5) and an outcome event incidence rate of 38.14%. Age (p=0.002), surgery (p<0.001), and overall survival (p<0.001) showed significant differences between the two datasets. All other p-values from statistical tests were greater than 0.05.

### 3.2 TAM calculation

The relative abundance of M2 macrophages was determined using the CIBERSORTx algorithm. The median values (interquartile ranges) for M2 macrophages were 4.78 (3.86-5.58) in the training set, 4.70 (3.76-5.63) in the validation set, and 4.84 (4.05-5.86) in the testing set, respectively. HCC patients were categorized into groups with high and low M2 macrophage content (M2group).

### 3.3 WSI classification performance

**Figure 2:**
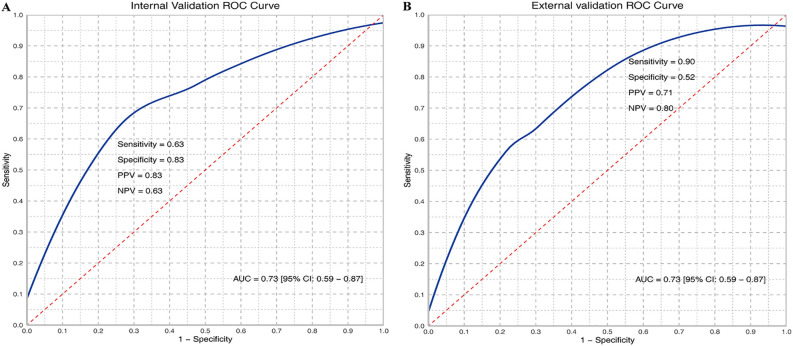
ROC curves of M2 relative abundance in internal **(A)** and external validation **(B)**. TP, True Positive; TN, True Negative; FP, False Positive; FN, False Negative; Sensitivity = TP/(TP + FN); Specificity = TN/(TN + FP); PPV = TP/(TP + FP); NPV = TN/(TN + FN); AUC, Area under the curve.

**Figure 3:**
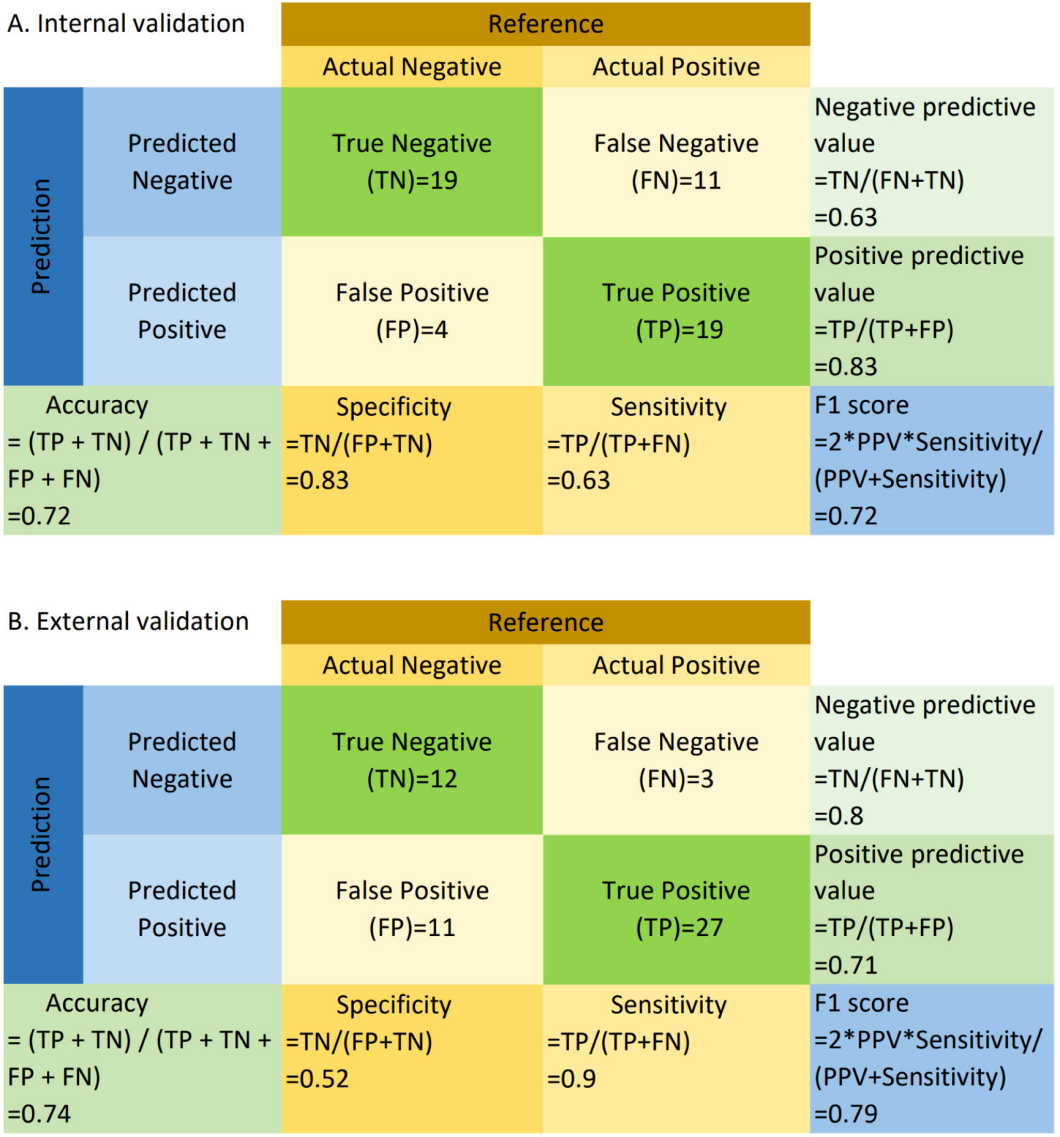
Confusion matrix for internal **(A)** and external validation **(B)**. TP, True Positive; TN, True Negative; FP, False Positive; FN, False Negative; Sensitivity = TP/(TP + FN); Specificity = TN/(TN + FP); PPV = TP/(TP + FP); NPV = TN/(TN + FN); Accuracy = (TP + TN)/(TP + TN + FP + FN); F1 = 2 * (PPV * Sensitivity)/(PPV + Sensitivity). “Negative” refers to cases where the relative abundance of M2 macrophages is less than or equal to the median, and “positive” refers to cases where the relative abundance of M2 macrophages exceeds the median.

Based on WSIs, we developed a model using the MAE approach to predict the relative proportion of M2 macrophages. In the validation dataset, the AUC (95% CI) was 0.73 (0.59-0.87), with sensitivity, specificity, PPV, and NPV values of 0.63, 0.83, 0.83, and 0.63, respectively. In the testing dataset, the AUC (95% CI) was 0.73 (0.59-0.87), with sensitivity, specificity, PPV, and NPV values of 0.90, 0.52, 0.71, and 0.80, respectively. ROC curves are shown in **Figures 2A** and **B**. The confusion matrices are presented in **Figure 3**.

### 3.4 Prognostic prediction

#### 3.4.1 M2prob and overall survival

We calculated predicted M2 macrophages proportion based on the WSIs. In the TCGA dataset, we included the M2 macrophages proportion, multiplied by 10 (M2prob), as an independent variable in the Cox regression equation to assess the impact of every 10% increase in M2 macrophages on prognosis. The results of the Cox regression analysis showed that M2 macrophage proportion was significantly associated with overall survival (HR=1.242, P=0.004), indicating statistical significance and suggesting that M2prob may influence the prognosis of liver cancer patients. Specifically, for every 10% increase in M2 macrophage proportion, the risk of death for patients increased by 24.2%. In the hospital dataset, M2prob remained significantly associated with prognosis (HR=1.89, P=0.031), with the risk of death for patients increasing by 89% for every 10% increase in M2 macrophage proportion. These results further supports its potential value as a prognostic factor.

#### 3.4.2 Mscore and overall survival

**Figure 4:**
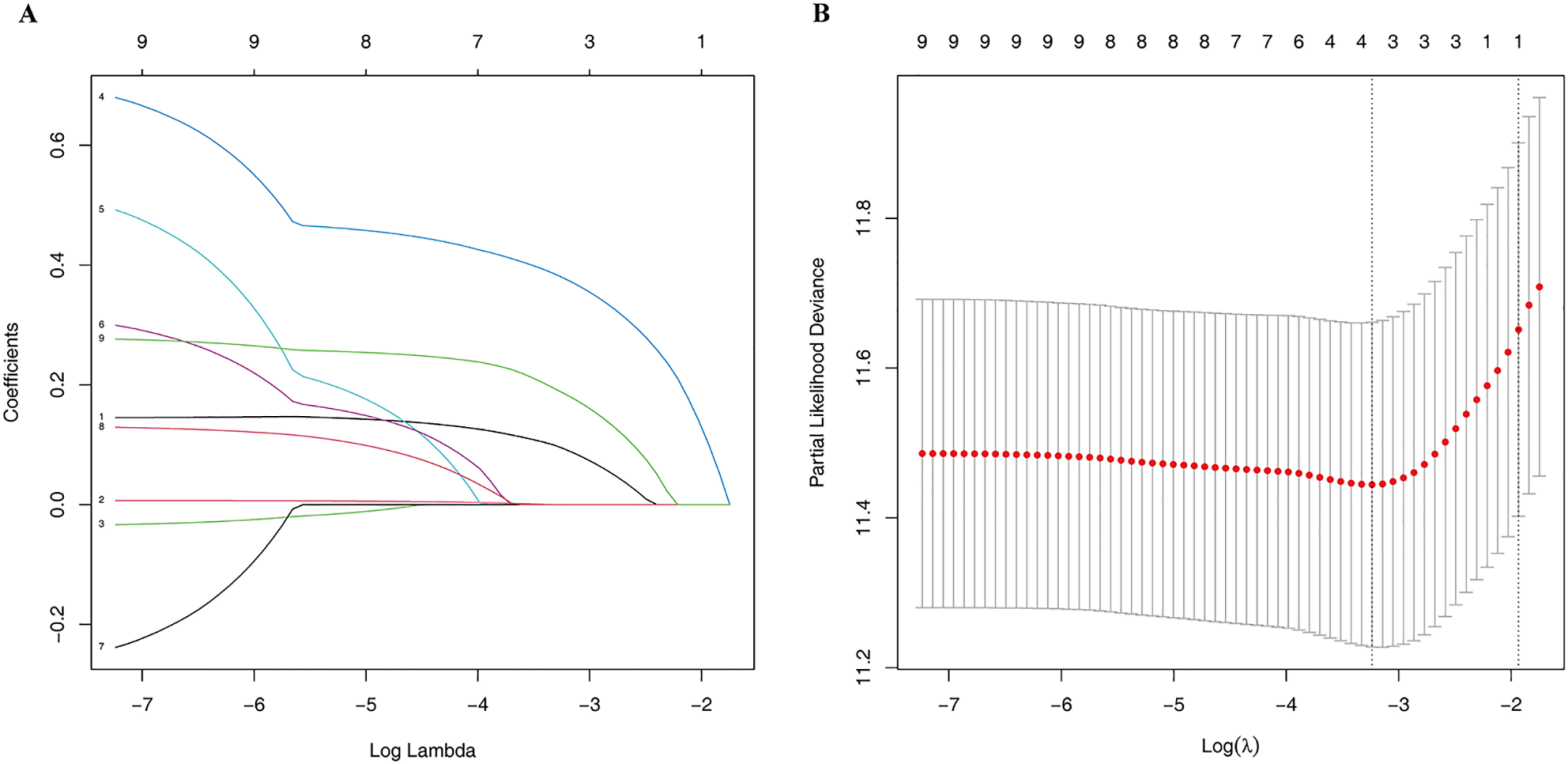
The Lasso regression process. The variation traits of the coefficient of factors **(A)** and the screening process of the Lasso regression model by 10-fold cross-validation **(B)**.

**Figure 5:**
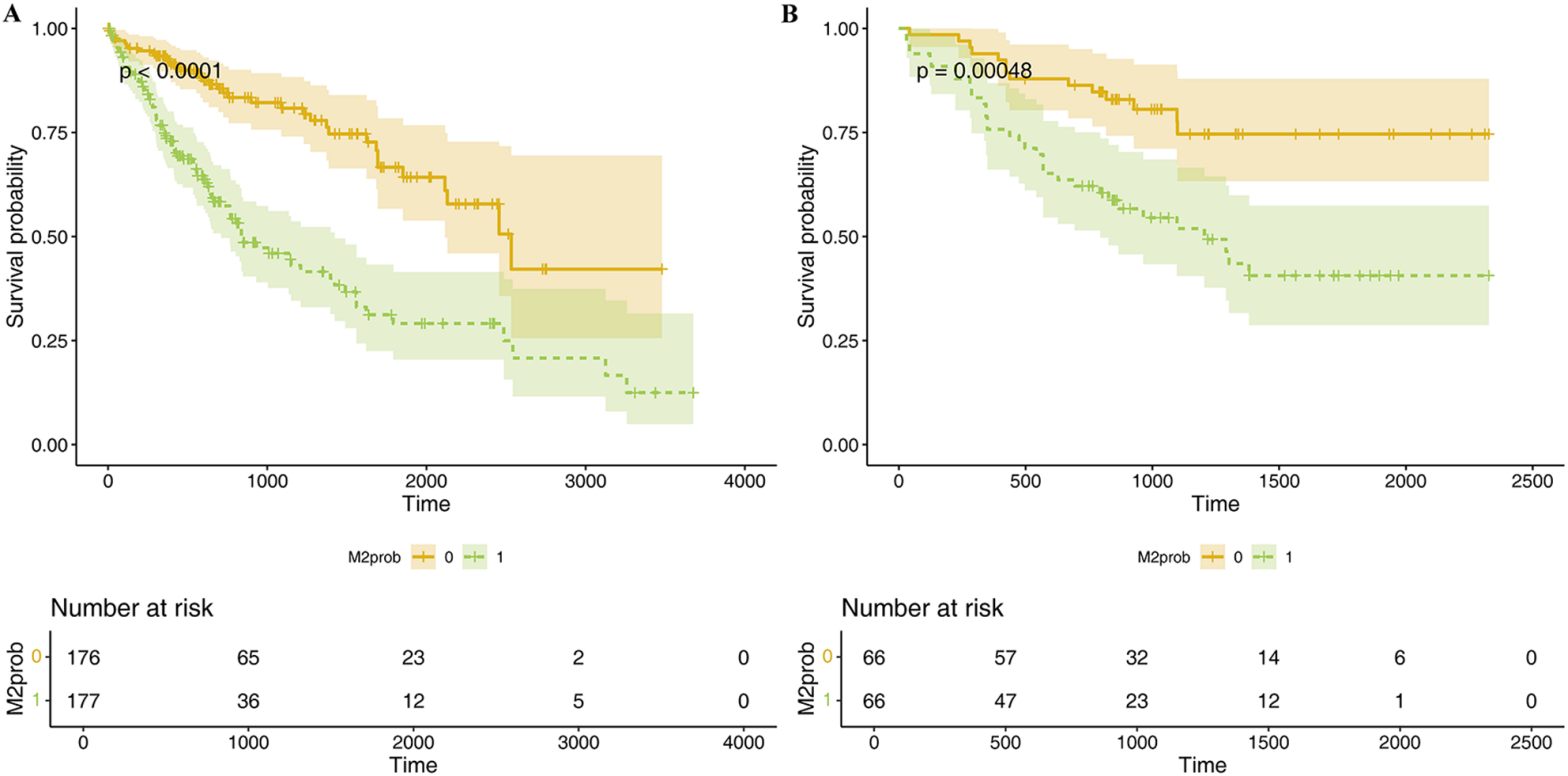
K-M survival curve of Mscore for predicting HCC prognosis in TCGA dataset **(A)** and hospital dataset **(B)**. The curves illustrate the survival differences between high and low Mscore groups, with statistical significance assessed by the log-rank test. Below each curve, the "number at risk" table is displayed, indicating the number of patients remaining at each time point in both groups.

Lasso regression was employed to identify risk factors. The coefficients of factors that finally enter the equation were shown in **Table 2**. **Figure 4A** showed the variation traits of the coefficient of factors. Ten-fold cross-validation method was used in the screening process, and a model with the smallest mean square error (MSE) were finally chosen (**Figure 4B**). Mscores were further calculated based on the screened risk factors and their coefficients in the Cox regression equation. The association between Mscore and survival prognosis in both the training and validation sets were calculated and Kaplan-Meier survival curves was constructed (**Figures 5A, B**).

**Table 2:** Multiple cox regression model in the training set.

	OR	95%CI	*P*
M2prob	1.186	1.021-1.377	**0.026**
t	1.594	1.324-1.919	**<0.001**
surgery	1.323	1.064-1.645	**0.012**

Rick factors were selected by Lasso regression analysis. Bold values indicate p-values less than 0.05.

## 4 Discussion

This study predicts M2 macrophage levels from WSIs using only global labels, and forecasts prognosis based on M2prob. To our knowledge, this is the first report of cell-level WSI prediction using the MAE-ResNet model. This study shows that using a deep learning method on WSIs can predict M2 macrophage levels. This capacity enables the prognosis of HCC patients without the need for additional clinical tests. Although further validation is needed through prospective studies, constructing an MAE-ResNet model shows promise for extracting vital clinical information from WSIs.

Tumor microenvironment plays a crucial role in tumor progression, comprising a diverse array of cells including tumor-associated macrophages, cancer-associated fibroblasts, endothelial cells, dendritic cells, B cells, T cells, and lymphocytes, etc. (16, 17) Macrophages are the most abundant of these (18). M2 macrophages, often called anti-inflammatory macrophages, are characterized by their production of anti-inflammatory mediators such as IL-10, transforming growth factor-beta, and arginase 1 (19). M2 macrophages are induced through several key signaling pathways. TGF-β binds to its receptors on macrophages, activating the PI3K/Akt/mTOR and TGF-β/Smad pathways. The PI3K/Akt/mTOR pathway regulates protein translation and transcription, while the TGF-β/Smad pathway controls gene expression by activating Smad proteins. IL-4 and IL-6 binding to their receptors trigger the JAK/STAT pathway, activating STAT3 and STAT6, which induce M2 gene expression. Notch ligand-receptor interactions activate the Notch pathway, leading to the expression of M2-related genes like Arg-1 and IL-10. The Wnt/β-catenin pathway also contributes by stabilizing β-catenin, which regulates genes involved in inflammation resolution. Hypoxia-inducible factor-2α (HIF-2α) plays a role in both the Notch and Wnt/β-catenin pathways under low-oxygen conditions, promoting an anti-inflammatory phenotype. Together, these pathways drive M2 macrophage polarization and contribute to their pro-tumorigenic functions, such as immune suppression and tissue remodeling (20). Specifically, M2 macrophages suppress the host’s anti-tumor immunity by increasing the expression of immunosuppressive surface proteins, producing reactive oxidants, secreting T cell inhibitory cytokines, and releasing chemokines that attract regulatory T cells, thereby diminishing the anti-tumor activity of effector T cells. Moreover, M2 macrophages could promote angiogenesis and enhance tumor invasion and metastatic potential through mechanisms such as VEGF and MMP (21).

Previous studies have shown that M2 macrophages are closely related to the prognosis of tumors. Regulating the polarization level through certain molecules can affect tumor progression. Research by Z. He et al. showed that exosome-derived FGD5-AS1 can promote the malignant behaviors of pancreatic cancer cells by promoting M2 polarization (22). Furthermore, the study by Rui Xu et al. highlights the significant role of M2 macrophages in HCC prognosis by identifying M2 macrophage-related genes through weighted gene co-expression network analysis (WGCNA), and establishing a 5-gene signature associated with immune infiltration for reliable prognostic assessment (23). In terms of disease treatment, trying to promote M1-type polarization or inhibit M2-type polarization to achieve anti-tumor purposes has also become a prominent research focus. Researches have been conducted on various tumors, such as ovarian cancer (24), Colorectal Cancer (25), Glioblastoma (26), etc. The research results of Bufu Tang et al. showed that xCT-specific knockout can limit HCC metastasis risk in transgenic mouse models by inhibiting M2-type polarization (27). There are also studies showing that Cancer treatment strategies that reprogram M2-like TAMs into M1-like cells have also yielded encouraging results (28).

In general, it is widely acknowledged that M2 macrophages facilitate tumor growth and migration (5, 29–32). Therefore, a convenient and effective method to predict M2 macrophage levels in daily diagnosis and treatment would provide a solid foundation for clinical decision-making and prognosis prediction. However, conventional detection methods like flow cytometry (33) and transcriptome-based CIBERSORTx continue to pose challenges in routine clinical diagnosis and treatment. In recent years, with the development of computer vision, the use of WSIs has broadened beyond pathology teaching and remote diagnosis (34). An increasing number of studies focused on directly diagnosing or classifying tumors through WSI. This research field, known as “computational disease” (35), is expected to profoundly change cancer diagnosis methods. Previous studies have demonstrated that deep learning methods using WSI can predict gene-level features (36–40) and cell-level features (41–43), thus, we believe extracting biomedical information from WSI holds significant research potential. Unfortunately, existing methodologies fall short in addressing the significant challenges presented by the high resolution of WSIs (44), a situation exacerbated by the shortage of comprehensively annotated datasets (45). In order to address this problem, numerous studies have segmented WSIs into patches and utilized the Multiple Instance Learning paradigm pretrained on ImageNet (9, 46, 47). However, models pretrained on ImageNet might not effectively identify distinctive medical features. In 2022, He et al. introduced a self-supervised learning method for computer vision called MAE (12). This method, which includes an encoder and a decoder, randomly masks parts of the input image and then reconstructs it.

In our study, we present a new MAE-ResNet modeling method, which uses the encoder part of MAE to extract WSI features, thus avoiding patch-level manual annotation work and predicting the relative abundance of M2 macrophage based only on slide-level labels. Our model offering the following advantages: (1) Compared to traditional CNN models, it eliminates the need for extensive work of manual annotation. The pixel size of WSIs is too large to be directly processed by CNN models under the constraints of the current GPU memory conditions. Traditional CNN models address this by dividing a WSI into thousands of smaller patches for annotation. However, this task is often unfeasible for busy pathologists, limiting many valuable studies in practice. The MAE-ResNet model we use, through a self-supervised autoencoder, eliminates this large-scale annotation workload. It employs an unsupervised approach by masking parts of the image and continuously attempting to predict the masked portions, thereby extracting image features. This innovative solution to the large pixel size problem eliminates the need for extensive annotation efforts. By simplifying the workload, it also paves the way for future studies with larger sample sizes. (2) Compared to traditional biological methods, such as flow cytometry and transcriptome-based CIBERSORTx, the model used in our study is more cost-effective and easier to implement in clinical settings, as it requires only routine clinical pathology sections and does not add extra workload or costs, providing a significant advantage for future clinical translation.

After developing the MAE-ResNet model to predict M2 macrophage levels, we further explored whether M2 macrophage levels and clinical characteristics could predict the prognosis of HCC patients. At this stage, data from the TCGA database were used for training and internal validation, while data collected from our hospital were used for external validation. The internal validation set (TCGA dataset) was used to assess the reproducibility of the model, while data from our hospital were used to evaluate the transportability of the initial model’s predictions. The following cox regression analysis indicated that the predicted probabilities of M2 macrophages was negatively associated with the prognosis of HCC. To enhance the generalizability of the model, we incorporated common covariates related to HCC prognosis into the predictive model. CIBERSORT deconvolution algorithm, weighted gene co-expression network analysis (WGCNA), and the LASSO algorithm are frequently used for dimensionality reduction (48), in this study, we employed the LASSO regression algorithm. Based on TAM prediction probability and clinical data, the prognostic prediction model we constructed also achieved satisfactory results in internal and external validation data sets, indicating good reproducibility and transportability for this model.

Despite the promising results of our study, several limitations must be acknowledged. First, ensuring the consistency and quality of pathological slides presents a significant challenge. To address this, we employed color standardization through histogram equalization during the image preparation stage, which helped reduce variations in color intensity and brightness caused by differences in slide sources or laboratory conditions. Additionally, our external validation set, sourced from Henan Provincial People’s Hospital, showed strong predictive performance. This may be partly due to the fact that the TCGA database contains slides from different institutions, which allowed our training set to learn the technical differences between these institutions. Another limitation is that the number of pathological images used in this study is relatively small, and a larger dataset is necessary to obtain more robust and generalizable results.

## Data Availability

The raw data supporting the conclusions of this article will be made available by the authors, without undue reservation.
